# New records of *Tylokepon* with the description of a new species (Epicaridea, Bopyridae, Keponinae)

**DOI:** 10.3897/zookeys.790.28134

**Published:** 2018-10-15

**Authors:** Jianmei An, Miao Zhang, Gustav Paulay

**Affiliations:** 1 School of Life Science, Shanxi Normal University, Linfen, 041000, PR China Shanxi Normal University Linfen China; 2 Florida Museum of Natural History, University of Florida, Gainesville, FL, 32611-7800, USA University of Florida Gainesville United States of America

**Keywords:** Bopyridae, Epicaridea, new records, new species, *
Tylokepon
*

## Abstract

The parasitic isopod genus *Tylokepon* is recorded for the first time from the Mariana Islands and Australia. *Tylokeponmarianensis***sp. n.** is described from the Mariana Islands, infesting *Thusaenysirami* (Laurie, 1906). The holotype female differs from other known *Tylokepon* females by the tri-lobed projection on pereomere 6, almost smooth lateral plates and pleopods, shape of oostegite 1, and widely opened brood pouch. The host is first recorded for bearing bopyrids. The new record of *T.bonnieri* Stebbing, 1904 from Australia on the type host extends the range of this species from China and India. A table of localities and hosts and a key to all species of *Tylokepon* are provided.

## Introduction

Epicarid isopods remain greatly underdescribed, and Williams and Boyko (2012) predicted that the central Indian Ocean and east Asian seas hold a wealth of undescribed species. The keponine genus *Tylokepon* Stebbing, 1904 is a case in point. It currently contains four species (An 2009), three of which are known from single collections. Here we add a fifth species and provide a new record for the only *Tylokepon* previously known from more than one record.

Boyko et al. (2013) recently erected the Keponinae for most genera previously attributed to Ioninae, restricting the latter to the type genus, *Ione* Latreille, 1818, and raising Ioninae to the family level. The keponine *Tylokepon* is readily differentiated from related genera by the prominent middorsal projections on the last two pereomeres and the extremely swollen bilobed head. Stebbing (1904) erected the genus for *T.bonnieri* Stebbing, 1904 infesting the epialtid crab *Tylocarcinusstyx* (Herbst, 1803) from the Maldives. An (2009) reported it from Beibu Gulf and South China Sea. Bonnier (1900) described *Ceponnaxiae* infesting *Hyastenusdiacanthus* (De Haan, 1839) from Hong Kong, based on a dried female specimen. After examining the type specimen, Markham (1982) transferred the species to *Tylokepon*. Shiino (1950) described a third species, *T.micippae*, infesting *Micippaphilyra* (Herbst, 1803) from Japan, and compared *Tylokepon* with the related genera *Grapsicepon* Giard & Bonnier, 1887, *Merocepon* Richardson, 1910, *Cancricepon* Giard & Bonnier, 1887, *Paracepon* Nierstrasz & Brender à Brandis, 1931, and *Scyracepon* Tattersall, 1905. An (2009) added a fourth species, *T.biturus*, infesting *Menaethiusmonoceros* (Latreille, 1825) from Hainan, China. Recorded hosts of *Tylokepon* are crabs belonging to the majoid families Epialtidae and Majidae, with a single host record from a species of Parthenopidae (An 2009).

In the present paper, two epialtid crabs bearing bopyrids were examined and the parasites identified as *T.bonnieri* and a new species, *T.marianensis*. These are new records for the genus from Australia and Micronesia. A table (Table 1) and a key to the species of *Tylokepon* are provided.

**Table 1.** Localities and hosts of all species of genus *Tylokepon* Stebbing, 1904.

Species	Localities	Hosts
*Tylokeponbiturus* An, 2009	China (Hainan)	*MenaethiusMonoceros* (Latreille, 1825)
*Tylokeponbonnieri* Stebbing, 1904	Indian Ocean (Maldives);	*Tylocarcinusstyx* (Herbst, 1803)
	China (Beibu Gulf and Hainan);	*Menaethiusmonoceros* (Latreille, 1825)
	Southwest India (Kovalam);	*Hyastenusdiacanthus* (De Haan, 1839)
	Australia (Queensland, Lizard Island)	*Enoplolambrusvalidus* (De Haan, 1837)
*Tylokeponnaxiae* (Bonnier, 1900)	Hong Kong	*Hyastenusdiacanthus* (De Haan, 1839)
*Tylokeponmicippae* Shiino, 1950	Japan	*Micippaphilyra* (Herbst, 1803)
*Tylokeponmarianensis* sp. n.	Mariana Islands, Guam Island	*Thusaenysirami* (Laurie, 1906)

## Material and methods

The material reported here was found infesting host decapods in the collections of the Florida Museum of Natural History, University of Florida (**UF**). Animals were viewed and drawn using a Zeiss Stemi SV Apo.

## Taxonomy

### Family BOPYRIDAE Rafinesque-Schmaltz, 1815

#### Subfamily Keponinae Boyko, Moss, Williams & Shields, 2013

##### Genus *Tylokepon* Stebbing, 1904

###### Tylokepon marianensis sp. n.

Taxon classificationAnimaliaIsopodaBopyridae

http://zoobank.org/4B1B0383-677A-4054-9230-60ECAC3462FF

Figure 1

####### Material examined.

UF 42220: holotype ♀and allotype♂, USA, Mariana Islands, Guam Island, Haputo, 13°34.74'N, 144°49.84'E, rubble, 8–10 m, 8 July 2003, Coll. G. Paulay, infesting the right branchial chamber of *Thusaenysirami* (Laurie, 1906) (host, UF 5935, identified by Dr. Amanda Windsor).

####### Diagnosis.

Female: Head large, swollen, and bilobed. Without eyes. Pereon with seven segments, sixth pereomere with tri-lobed projection and seventh pereomere with a single, large median projection. Pleon of six segments, first five with uniramous lateral plates and biramous pleopods. Lateral plates tuberculated on both sides of first pleomeres, but smooth on remaining pleomeres. Sixth pleomere small with uniramous uropoda.

Male: Head semicircular, with black eyes. Seven pereomeres with truncate margins. Pereopods subequal in size and structure. First five pleomeres with tuberculate, uniramous pleopods. Sixth pleomere with a pair of round uropods covered in scales and each ramus with stout terminal setae.

####### Description of holotype female.

**Figure 1.**
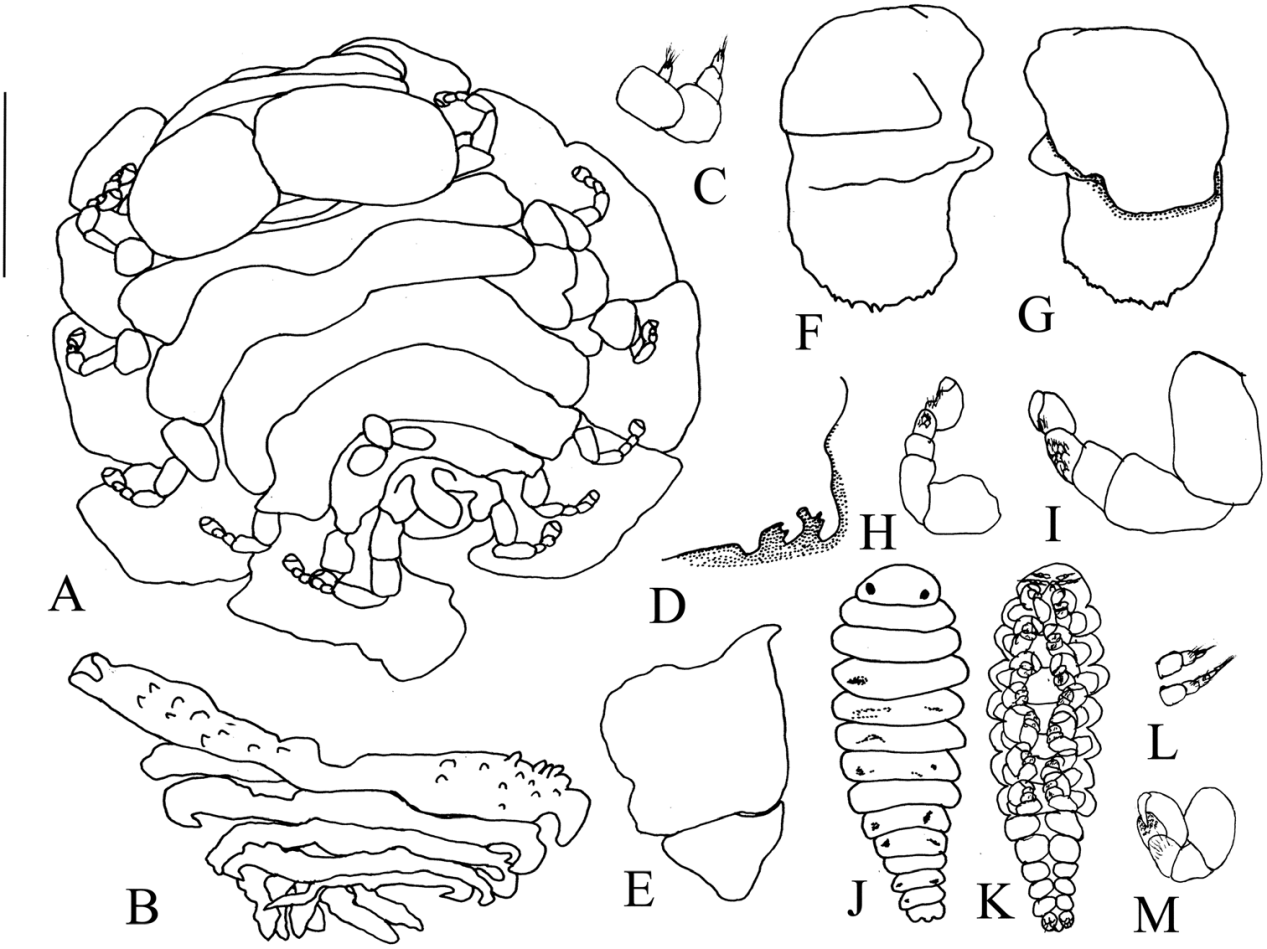
*Tylokeponmarianensis* sp. n. Holotype female (UF 42220) (**A–I**). **A** Dorsal view **B** Pleon, dorsal view **C** Left antennae **D** Barbula, left side **E** Right maxilliped, external view **F** Left oostegite 1, external view **G** Left oostegite 1, internal view **H** Left pereopod 1 **I** Left pereopod 6. Allotype male (**J–M**) **J** Dorsal view **K** Ventral view **L** Left antenna **M** Left pereopod 4. Scale bars: 1.00 mm (**A, B**); 0.63 mm (**D–G**); 0.35 mm (**H, I, L, M**); 0.50 mm (**J, K**).

Length 3.38 mm (excluding pleon and uropods), maximum width 1.9 mm across pereomere 4, head length 0.73 mm, head width 1.5 mm. All body regions and segments distinct; no pigmentation (Figure 1A, B).

Head large, covering pereomere 1, wider than long, bilobed, comprised of two ellipsoid structures separated by a deep median groove. Frontal lamina narrow and not extending to margin of head. Eyes absent (Figure 1A). First and second antennae rudimentary with two and three articles, respectively, terminally setose (Figure 1C). Barbula (Figure 1D) with two lateral digitate projections and a medial blunt projection on each side. Maxilliped (Figure 1E) with a smooth, curved palp and a sharp plectron; anterior article much larger than posterior one.

Pereon broadest across pereomere 3. Head covering much of pereomere 1, with only median part visible in dorsal view. Dorsolateral bosses distinct on first four pereomeres on both sides. Coxal plates absent. Tergal projections present on pereomeres 2–4. Last two pereomeres with middorsal projections: pereomere 6 with a tri-lobed projection; pereomere 7 with a single, large median projection (Figure 1A). Brood pouch widely open. Oostegite 1 (Figure 1F, G) composed of two equally-sized articles, internal ridge smooth, jagged posterior margin with sharp projections, without posterolateral point. Pereopod 1 much smaller than others, but all pereopods subequal in shape, all articles distinct, carpi and propodi with setae on ventral surface (Figure 1H, I).

Pleon with six distinct pleomeres, first five with longer lateral plates and biramous pleopods (Figure 1B). Lateral plates tuberculated on both sides of first pleomeres, but smooth on remaining pleomeres. Terminal segment without lateral plates. All pleopods with slightly undulating to smooth margins, gradually shorter toward posterior. Pleomere 6 small. Uropods uniramous, lobose, with smooth surfaces and entire margins (Figure 1B).

####### Description of allotype male.

Length 0.95 mm, maximal width 0.23 mm across pereon 3, head length 0.11 mm, head width 0.19 mm, pleonal length 0.31 mm. All body segments distinct with scattered pigmentation (Figure 1J, K).

Head semicircular, black eyes near posterior margin of head (Figure 1J). First and second antennae of three and four articles, respectively (Figure 1L), terminal articles fringed with setae. Third pereomere widest; all pereomeres with truncate margins. Pereopods subequal in size and structure (Figure 1M), meri and carpi with setae on ventral surfaces, each pointed dactylus retracts into groove formed between parallel series of tubercles on each propodus.

Pleon of six segments, first five pleomeres with tuberculate, uniramous pleopods. Sixth pleomere with a pair of round uropods covered in scales and each ramus with stout terminal setae; medial anal cone smooth.

####### Etymology.

The specific epithet, *marianensis*, refers to the type locality in the Mariana Islands.

####### Remarks.

**Figure 2.**
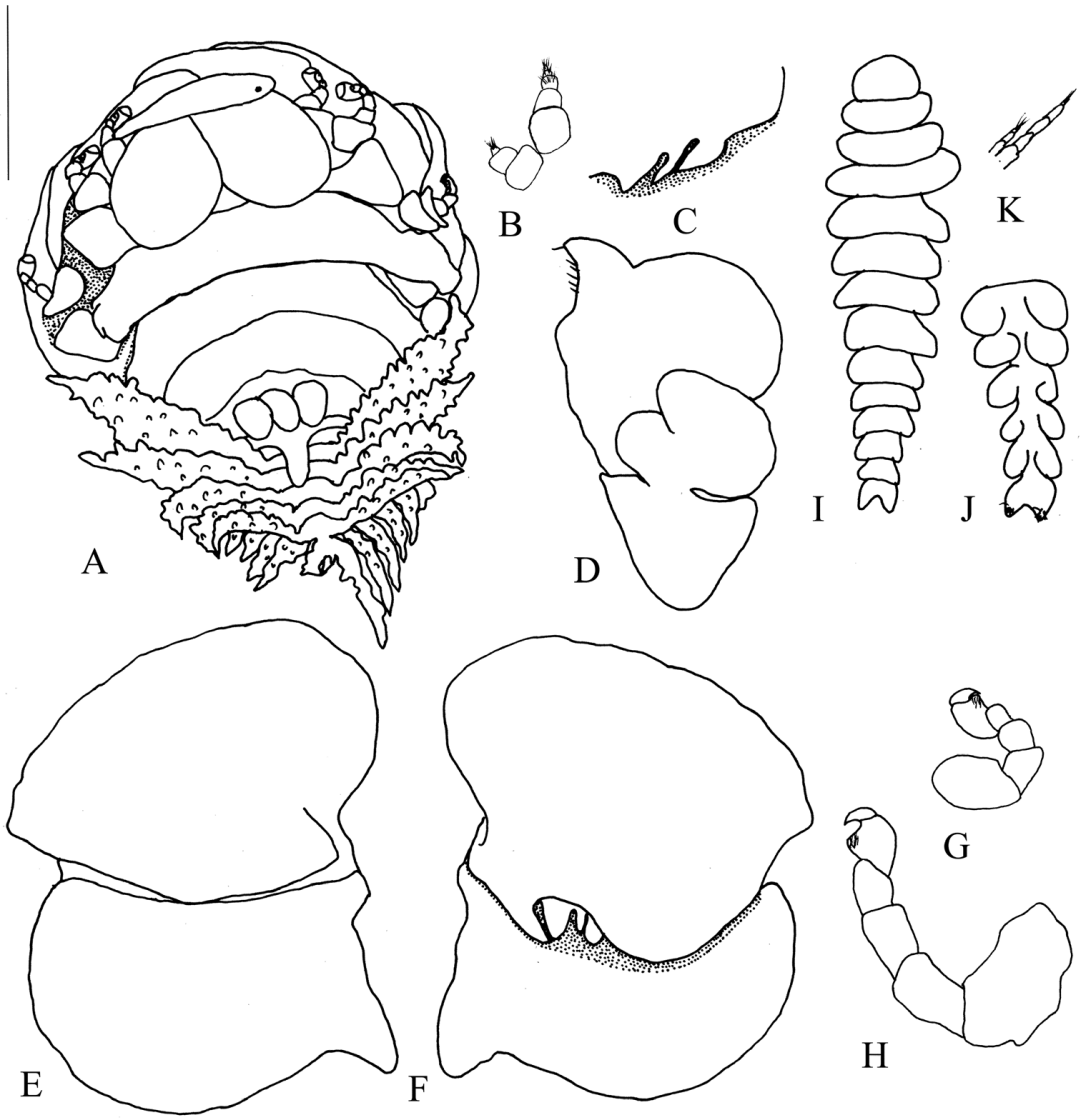
*Tylokeponbonnieri* Stebbing, 1904. Female (UF 42219) (**A–H**). **A** Dorsal view **B** Right antennae **C** Barbula, left side **D** Left maxilliped, external view **E** Left oostegite 1, external view **F** Left oostegite 1, internal view **G** Right pereopod 1 **H** Left pereopod 5. Male (UF 42219) (**I–K**). **I** Dorsal view **J** Left antenna **K** Pleon, ventral view. Scale bars: 1.00 mm (**A**); 0.65 mm (**C**); 0.42 mm (**D–F**); 0.31 mm (**B, G, H, J**); 0.42 mm (**I, K**).

Shiino (1950) and Markham (1982) summarized the distinguishing features of *Tylokepon* as follows: females have a large head formed by two semi-spherical structures separated by a deep median groove, and the last two pereomeres possess prominent middorsal projections. The present specimens are referred to *Tylokepon* on the basis of these characters. The new species is distinguished from the other four nominal species of *Tylokepon* by the tri-lobed middorsal projection on the sixth pereomere of females and the jagged posterior margin of oostegite 1. *Tylokeponmarianensis* is most similar to *T.bonnieri*, which was recorded from Beibu Gulf in China on the related host *Hyastenusdiacanthus* (An, 2009). Both have the middorsal projections on pereonite 6 consisting of three lobes and a single projection on pereonite 7. However, while the three lobes are separated at the base in *T.bonnieri*, they are united basally to form a tri-lobed structure in the new species (compare Figs 1A and 2A). They also differ in the sculpture of the lateral plates on pleomeres 2–5 and pleopods: these are almost smooth in *T.marianensis* but tuberculate in *T.bonnieri*. *Tylokeponmicippae* differs from *T.marianensis* in having a closed, rather than widely open brood pouch, tuberculate rather than nearly smooth pleopods and lateral plates on pleomeres 2–5, a sharp and pointed posterolateral point on oostegite 1 rather than an entire margin, and a cluster of three rather than a single mid-dorsal projection on the seventh pereomere. The poorly known *T.naxiae*, which also infests *H.diacanthus*, differs from the new species in the shape of the middorsal projections of the sixth pereomere, which are separated at the base as in *T.bonnieri*, and having digitate, rather than entire margins on the lateral plates of the second pleomeres. The new species also differs from *T.biturus* in having three rather than two mid-dorsal projections on the sixth pereomere, having the lobes of the head united into a dumbbell shape rather than separated by a groove, and smooth, rather than tuberculate, lateral plates on the first pleomeres.

###### Tylokepon bonnieri

Taxon classificationAnimaliaIsopodaBopyridae

Stebbing, 1904

Figure 2

Tylokepon bonnieri Stebbing, 1904: 716–717, pl. LIII, B, C; Shiino 1950: 166; Pillai 1954: 19; Pillai 1964: 187–188, figs. 7–11; An 2009: 96–98.

####### Material examined.

UF 42219: 1♀, 1♂, Australia, Queensland, Lizard Island, north side, at “Washing Machine, 14°39.02'S, 145°27.73'E, from dead *Pocillopora*, 21 February 2009, coll. Molly Timmers. Infesting right branchial chamber of *Tylocarcinusstyx* (Herbst, 1803) (UF 18294).

####### Description of female.

Length 3.58 mm (excluding uropods), maximum width 2.28 mm across pereomere 3, head length 0.66 mm, head width 1.26 mm. (Figure 2A).

Head large, covering pereomere 1, wider than long, completely bilobed, with two ellipsoid structures separated by a deep median groove. Frontal lamina narrow, visible in dorsal view. Small black eyes near frontal lamina (Figure 2A). First and second antennae rudimentary, with three and five articles, respectively, terminally setose (Figure 2B). Barbula (Figure 2C) with two large falcate projections and a mediad sharp projection on each side. Anterior article of maxilliped (Figure 2D) much larger than posterior one, with prominent, inwardly-directed and setose palp, plectron short.

Pereon broadest across pereomere 3. Head covering much of pereomere 1. Distinct dorsolateral bosses on first four pereomeres on both sides. Coxal plates absent. Tergal projection present on pereomeres 2 and 3. Middorsal projections on last two pereomeres: three parallel projections on pereomere 6, one large and posteriorly extended projection on pereomere 7 (Figure 2A). Brood pouch incompletely covered by oostegites. Oostegite 1 (Figure 2E, F) composed of two subequal in size articles, with smooth internal ridge that bears three marginal projections; ending in extended posterolateral point. Pereopod 1 much smaller than others, all pereopods with short dactyli (Figure 2G, H).

Pleon of six distinct pleomeres, first five with well-developed, tuberculate lateral plates, and biramous, tuberculate pleopods. All lateral plates and exopodites of pleopods with digitate margins. Endopodites of pleopods 1–5 small and smooth (Figure 2A). Uniramous uropods similar to exopodites of pleopod 5.

####### Description of male.

Length 1.08 mm, maximal width 0.32 mm, across pereomere 3, head length 0.11 mm, head width 0.16 mm, pleonal length 0.39 mm. Body gradually tapered posteriorly, all segments distinct (Figure 2I).

Head semicircular, broader than long; without eyes. First and second antennae of three and five articles, respectively (Figure 2J), basal articles greatly expanded, final articles fringed with setae. Pereon broadest across pereomere 3. Pleon of six segments, first five pleomeres with tuberculate uniramous pleopods. Pleomere 6 bilobed, with tuberculate projections and setae (Figure 2K).

####### Remarks.

Stebbing (1904) erected *Tylokepon* for *T.bonnieri* infesting *Tylocarcinusstyx* from the Indian Ocean. Pillai (1954, 1966) recorded the species from Kovalam, southwest India, infesting *Menaethiusmonoceros*. An (2009) reported it from Beibu Gulf and Hainan, China, infesting *Hyastenusdiacanthus* and the parthenopid crab *Enoplolambrusvalidus* (De Haan, 1837). Although *Hyastenusdiacanthus* was recorded to be infested by *Tylokeponnaxiae* from Hong Kong, it is not impossible that a single host is found bearing two different species, such as the case of *Petrolishthesquadratus* Benedict, 1901 bearing *Aporobopyruscrutatus* (Richardson, 1910) and *A.bonairensis* Markham, 1988.

As for *T.naxiae*, Bonnier (1900) only gave a brief description, and Markham (1982) found that the holotype female is badly damaged, and only three useful characters can be defined. However, from the figure (Markham 1982: fig. 18), it can be seen that the species has an almost smooth surface to the pleopods and the lateral plates are not covered with tuberculate (while the pleopods and lateral plates of *T.bonnieri* are densely covered with tubercles). Therefore, *T.bonnieri* differs from *T.naxiae*.

The present specimens conform well to Stebbing’s description (1904), except for some minor differences, such as the male specimen lacking eyes. There are slight differences between the present specimens and those from Beibu Gulf (An 2009) in the middorsal projections on pereopod 6 and frontal lamina of the head, but the key characters of the females, such as oostegite 1, maxilliped, barbula, lateral plates and pleopods, as well as of males, are similar.

##### Key to species of *Tylokepon*

**Table:** 

1	Three middorsal projections on pereomere 6	**2**
–	Two middorsal projections on pereomere 6	***T.biturus* An, 2009**
2	Lateral plates of all pleomeres with digitate margins	**3**
–	Lateral plates of pleomeres 2–5 nearly smooth	***T.marianensis* sp. n.**
3	Two lobes of head separated by a shallow median groove	***T.micippae* Shiino, 1950**
–	Two lobes of head completely divided by deep median groove	**4**
4	Ventral surface of pleopods almost smooth	***T.naxiae* (Bonnier, 1900)**
–	Ventral surface of pleopods densely tuberculated	***T.bonnieri* Stebbing, 1904**

## Supplementary Material

XML Treatment for Tylokepon marianensis

XML Treatment for Tylokepon bonnieri
